# Elevation in viral entry genes and innate immunity compromise underlying increased infectivity and severity of COVID-19 in cancer patients

**DOI:** 10.1038/s41598-021-83366-y

**Published:** 2021-02-25

**Authors:** Jennifer Yin Yee Kwan, Liang-Tzung Lin, Rachel Bell, Jeffrey P. Bruce, Christopher Richardson, Trevor J. Pugh, Fei-Fei Liu

**Affiliations:** 1 Department of Radiation Oncology, University of Toronto, Toronto, ON Canada; 2 Institute of Medical Science, University of Toronto, Toronto, ON Canada; 3 Department of Microbiology and Immunology, School of Medicine, College of Medicine, Taipei Medical University, Taipei, Taiwan; 4 Graduate Institute of Medical Sciences, College of Medicine, Taipei Medical University, Taipei, Taiwan; 5 Princess Margaret Cancer Centre, Toronto, ON Canada; 6 Department of Microbiology and Immunology, Faculty of Medicine, Dalhousie University, Halifax, NS Canada; 7 Department of Pediatrics, Faculty of Medicine, Dalhousie University, Halifax, NS Canada; 8 Ontario Institute for Cancer Research, Toronto, ON Canada; 9 Radiation Medicine Program, Princess Margaret Cancer Centre, 700 University Avenue, Toronto, ON M5G 2M9 Canada; 10 Department of Medical Biophysics, University of Toronto, Toronto, ON Canada

**Keywords:** SARS-CoV-2, Cancer

## Abstract

Multiple studies have reported a doubling in risk of Coronavirus Disease-2019 (COVID-19) among cancer patients. Here, we examine the potential biological rationale behind this recurrent epidemiological observation. By leveraging large-scale genome-wide transcriptional data of normal and malignant tissues from adults and children, we found evidence of increased expression of SARS-CoV-2 viral entry genes in the cancer state, particularly in respiratory, gastrointestinal, and genitourinary tract tissues, with decreased expression in pediatric *vs*. adult samples. Additionally, by interrogating the temporal effects of radiotherapy on human peripheral blood mononuclear and mucosal cells, we observed important treatment-related alterations in host innate immunity, specifically type I interferon responses. Overall, cancers enhance expression of critical viral entry genes, and innate viral defenses can be dysregulated transiently during radiation treatments. These factors may contribute to the observed increased susceptibility to SARS-CoV-2 entry and severity of COVID-19 in cancer patients.

## Introduction

Currently, cancer patients comprise a greater than expected proportion of all patients who have Coronavirus Disease-2019 (COVID-19). Initial studies in Wuhan, China reported that approximately 1 in 14 hospitalized COVID-19 patients had cancer^1^. A subsequent nationwide analysis in China reported that 18 out of 1,590 COVID-19 cases had a medical history of cancer, with an incidence of 1% compared to 0.29% in the general population^2^. Furthermore, the odds ratio of a cancer patient being infected compared to other members of the community has been reported to be 2.31 (95% CI 1.89–3.02)^3^. A systematic review and meta-analysis showed the pooled prevalence of cancer was 3.50% (95% CI 1.70–5.80) and pooled risk ratio for a cancer patient to develop severe disease to be 1.76 (95% CI 1.39–2.23) across 20 studies and 32,404 patients from the United States, United Kingdom, Italy, Singapore, Thailand, France, India, South Korea, as well as China^4^. These data are corroborated by our own institutional experience, whereby routine COVID testing for all cancer patients during March to June 2020 showed a positivity rate of 0.83% (29/3,491), on a background of 2,191 active COVID-19 cases in a metropolitan population of 2.9 M in Toronto, Canada (0.08%)^5,6^.

**Figure 1:**
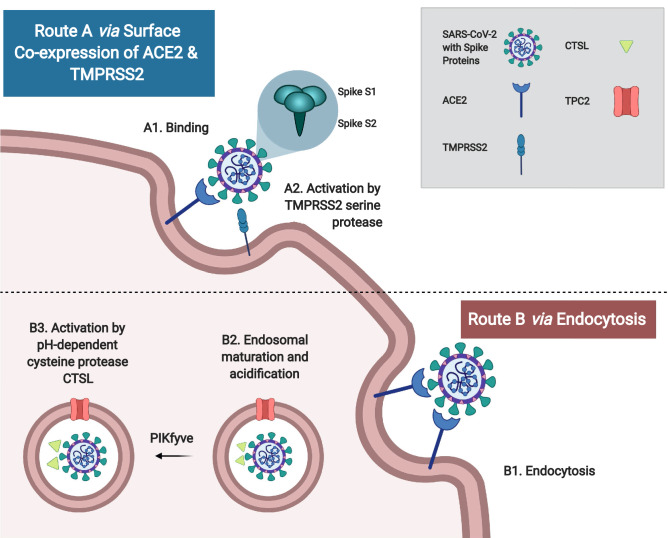
Routes of coronavirus entry. Severe acute respiratory syndrome coronavirus-2 (SARS-CoV-2) contains a transmembrane spike (S) glycoprotein, which is important for (i) host cell receptor binding by the S1 subunit, and (ii) subsequent S2 subunit activation via S protein proteolytic cleavage to mediate virus-cell fusion and entry. There are two major routes of viral activation: **Route A:** When angiotensin-converting enzyme 2 (ACE2) and transmembrane protease serine 2 (TMPRSS2) are co-expressed on the host cell surface, SARS-CoV-2 will bind to ACE2 and become activated by TMPRSS2 via proteolytic cleavage to mediate viral-cell fusion. **Route B:** If there is no surface expression of a protease, SARS-CoV-2 can undergo endocytosis, endosomal maturation (mediated by two pore channel subtype 2 (TPC2) and phosphatidylinositol 3-phosphate 5-kinase (PIKfyve)), followed by cleavage with pH-dependent cysteine protease cathepsin L (CTSL). Figure created with BioRender.com.

Characteristically, coronavirus (CoV) infection begins with viral entry that is mediated by the transmembrane spike (S) glycoprotein. This process entails (i) host cell receptor binding by the S1 subunit, and (ii) proteolytic cleavage to yield the activated S2 subunit, which mediates virus-cell fusion and entry at the endosomes^7^ (Fig. 1). Similar to the closely related SARS-CoV, SARS-CoV-2, the novel coronavirus responsible for COVID-19, uses the angiotensin-converting enzyme 2 (ACE2) type I membrane protein as its primary receptor in cross-species and human-to-human transmission, which occurs primarily via respiratory droplets and contact^7–12^. In addition to the respiratory tract and lungs, human ACE2 is broadly expressed in many organs including the heart, kidney and intestine. This is consistent with non-respiratory symptoms in COVID-19 patients such as acute cardiac injury, renal failure, and diarrhea^9,13–15^. A risk map based on ACE2 expression has identified the respiratory tract, esophagus, lung, heart, kidney, ileum and bladder as highly vulnerable organs^13^. Specifically, there is enriched ACE2 expression in type II alveolar cells (AT2), myocardial cells, proximal tubules of the kidney, ileum and esophageal epithelial, and bladder urothelial cells^13^. In addition to ACE2, host cell serine protease transmembrane protease serine 2 (TMPRSS2) is also important for the S glycoprotein activation for fusion^16^. Unlike SARS-CoV, the SARS-CoV-2 spike protein contains a polybasic cleavage site at the S1/S2 junction, which can be cleaved by furin, leading to efficient cell-to-cell transmission, possibly increasing COVID-19 disease severity^17^. Other important host cell factors that contribute to SARS-CoV-2 entry include phosphatidylinositol 3-phosphate 5-kinase (PIKfyve), lysosomal two pore channel subtype 2 (TPC2), and endosomal cysteine protease cathepsin L (CTSL)^11^. Overall, a number of factors facilitate entry of SARS-CoV-2 during COVID-19; amongst these, ACE2, TMPRSS2, and CTSL have been repeatedly identified as central viral entry factors, as summarized in Fig. 1.

COVID-19 can range from asymptomatic and mild disease in most cases (~ 80%) to severe respiratory dysfunction and critical multi-organ failure (~ 20%), with an overall fatality rate of 2–3%^18^. COVID-19 can progress in patients through three stages, beginning with an initial 2- to 14-day incubation period, with or without detectable virus, during which time asymptomatic transmission can occur. The second stage consists of a non-severe symptomatic period with detectable levels of virus^19^. Symptoms can be more evident, characterized by fever, fatigue, cough and shortness of breath, with many patients developing lymphopenia and pneumonia^9,18^. Initially, effective restriction of severe disease progression by the host relies primarily on innate immunity and the type I interferon (IFN) response that can be triggered by pathogen associated molecular patterns (PAMPs) recognition^18^. Type I IFN robustly activates antiviral defense via over 200 IFN-stimulated genes (ISGs), including IFN-induced transmembrane protein 3 (IFITM3)^20^, MX dynamin like GTPase 1 (MX1)^21^, tripartite motif containing 25 (TRIM25)^22^, and SAM and HD domain containing deoxynucleoside triphosphate triphosphohydrolase 1 (SAMHD1)^23,24^, which suppress viral replication and/or spread, thereby promoting clearance of infected cells^18,25,26^. In contrast, the virus expresses proteins that can delay or inhibit the early type I IFN response. Deregulation of innate immunity can lead to a hyperinflammatory response with over-production of cytokines such as IL-2, IL-7, IL-10, G-CSF, IP-10, MCP-1, MIP-1A, and TNF-α^18^. Indeed, SARS-CoV-2 is sensitive to type I IFN pre-treatment *in vitro*^27,28^; however, it is found to produce low type I and III IFN responses in vivo in ferrets and COVID-19 patients^28^. COVID-19 patients exhibit increased production of neutrophils, increased levels of serum IL-6, C-reactive protein, and decreased numbers of lymphocytes, correlating with more severe disease requiring intensive care^12,14,18^. Cytokine storm induction is thought to drive disease progression into stage three, severe respiratory disease with viral sepsis, causing acute respiratory distress syndrome (ARDS), respiratory failure, and potentially multi-organ failure leading to death^18,19^.

Based on the recurrent trend of increased rates of infection and severity in oncology patients reported across multiple studies as well as our own experience, this paper leverages large-scale genome-wide transcriptional data of normal and malignant tissues from human adults and children to illustrate increased expression of viral entry genes in the cancer state, particularly in respiratory, gastrointestinal, and genitourinary tract tissues, as well as decreased expression in pediatric *vs.* adult samples. Additionally, by interrogating the effects of cancer radiotherapy and chemotherapy on human tissues, biological pathways of innate immunity are identified to be commonly dysregulated by both cancer treatments and COVID-19.

## Results

### ACE2, TMPRSS2, and CTSL expression varies across normal tissues of the human body

**Figure 2:**
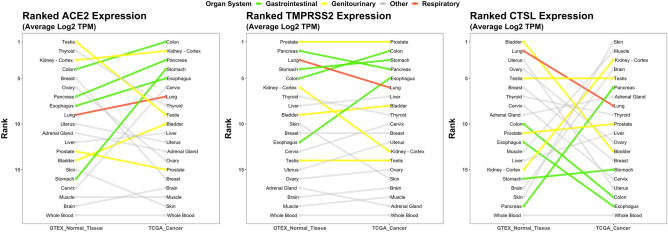
RNA expression of ACE2, TMPRSS2, and CTSL in normal and malignant tissues. RNA expression of *ACE2 (left)*, *TMPRSS2 (middle)*, and *CTSL (right)* in 20 matched normal and malignant tissues from the Genotype-Tissue Expression Portal (GTEx, n = 4,744 Samples)^29^ and The Cancer Genome Atlas (TCGA, n = 9,026 Samples)^30^; respectively. The mean expression in log_2_ TPM (transcripts per million) was calculated for each tissue, then ranked based on each gene. Organ systems of interest are highlighted by colour: gastrointestinal tract (green), genitourinary tract (yellow), respiratory tract (red), and other organ systems (grey).

RNA expression of three critical SARS-CoV-2 entry genes (*ACE2, TMPRSS2*, and *CTSL*) were analyzed across 20 different normal tissues from the Genotype-Tissue Expression (GTEx) Portal^29^. Normal tissues spanned the human body from head to toe and covered all major organs and tissue types including the central nervous system, gastrointestinal tract, genitourinary tract, breast and gynecological organs, respiratory tract, endocrine system, muscle, skin, and blood. The mean expression in log_2_ (transcripts per million) of each viral entry gene was calculated for each tissue and ranked from highest to lowest (Fig. 2). The top expressing tissue for all three of the critical viral entry genes were observed in the normal genitourinary tract (testis for *ACE2*, prostate for *TMPRSS2*, and bladder for *CTSL*). Other high expressing tissues were observed in the gastrointestinal tract (colon, pancreas, stomach); of note, brain, muscle, and blood were among the lowest expressing tissues.

### Malignancy increases expression of viral entry genes

Different cancer types were then examined for their association with expression of viral entry genes. RNA expression of *ACE2*, *TMPRSS2*, and *CTSL* were analyzed across 20 malignant tissues from The Cancer Genome Atlas (TCGA)^30^. The top cancers expressing *ACE2* were from the gastrointestinal (colon, pancreas, stomach, and esophagus), and genitourinary tracts (kidney) with each of these cancers having increased rank expression compared to their matched normal tissue counterparts (Fig. 2, Supplementary Table S1). The top cancers expressing *TMPRSS2* were also from the gastrointestinal (colon, stomach, pancreas, and esophagus) and genitourinary (prostate) tracts. Four of these five cancer types were the same as the top expressing cancers for *ACE2*. *CTSL* expressing cancers had varied rank expression with skin, muscle, kidney, brain, and testis being the top expressing cancers. Of note, many of the top ranked normal tissues for *CTSL* expression were among the bottom-ranked malignant tissues for *CTSL* expression, and vice versa with cancer type having a large effect on *CTSL* tissue expression rank.

### Pediatric tissues have lower expression of viral entry factors

**Figure 3:**
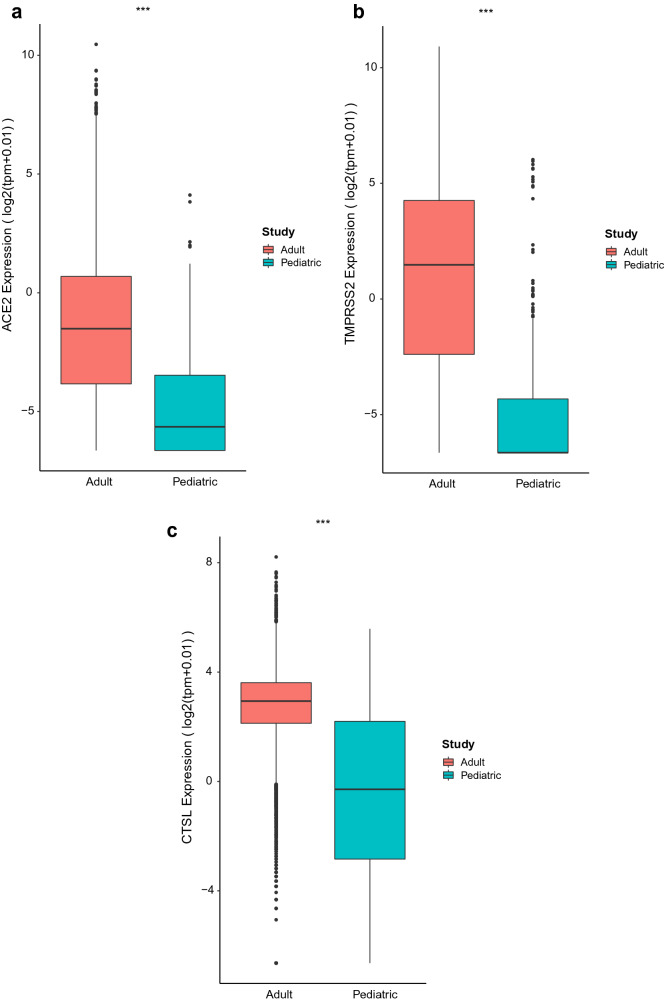
ACE2, TMPRSS2, and CTSL expression in adult and pediatric malignancies. Comparison of median (**a**) *ACE2*, (**b**) *TMPRSS2*, and (**c**) *CTSL* expression from 7 pediatric cancer tissues from TARGET (n = 734), and 33 cancer tissues from The Cancer Genome Atlas (TCGA, n = 10,535) was conducted. Gene expression is expressed as log_2_-transformed function of TPM. ANOVA with Tukey’s test was performed for comparison of expression between studies. ***p < 0.001.

The most common childhood cancers are hematologic and central nervous system malignancies. *ACE2, TMPRSS2*, and C*TSL* expression were compared in children (TARGET, n = 7 pediatric cancers) *vs*. adult tissues (TCGA dataset, n = 33 adult cancers)^31,32^. Indeed, all viral entry genes were observed to have significantly lower expression in pediatric compared to adult cancer types (Fig. 3; *p* < 0.001).

### Sex and smoking alter expression of viral entry genes

**Figure 4:**
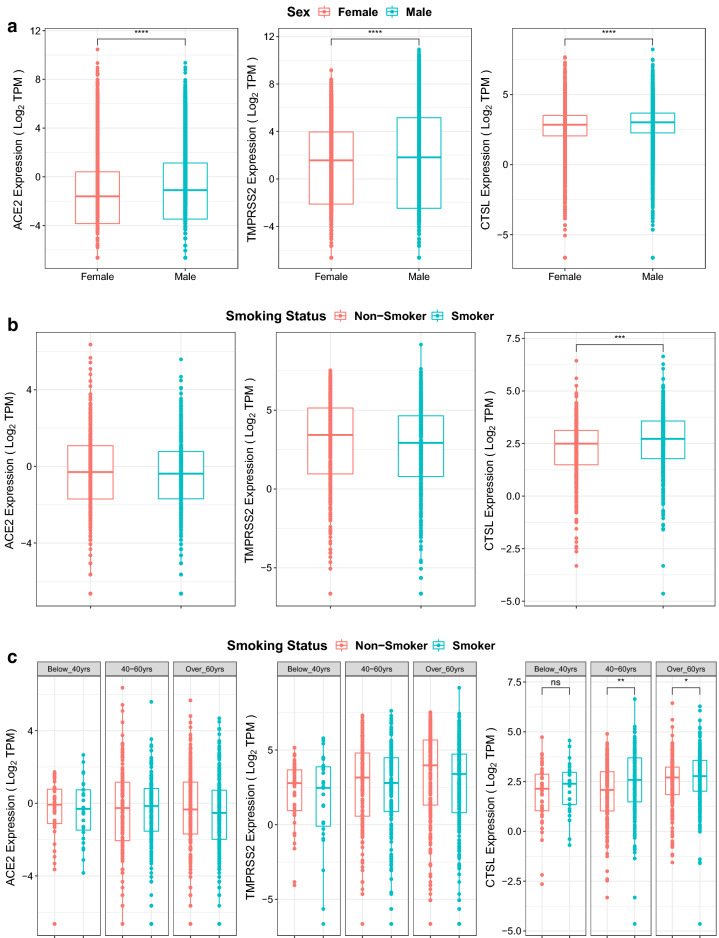
Effect of smoking and sex on ACE2, TMPRSS2, and CTSL expression. (**a**) Expression of *ACE2 (left)*, *TMPRSS2 (middle)*, and *CTSL (right)* in TCGA data for males and females for 33 cancer types (n = 12,736) with available annotations in TCGA. Wilcoxon signed rank tests were performed and significant differential expression between groups was labelled ****p < 0.0001 between males and females. (**b**) Expression of *ACE2 (left)*, *TMPRSS2 (middle)*, and *CTSL (right)* in TCGA data for non-smokers and smokers with lung squamous cell carcinoma, lung adenocarcinoma, cervical cancer, bladder urothelial carcinoma or esophageal carcinoma (n = 873). Wilcoxon signed rank tests were performed and significant differential expression between groups was labelled. ***p < 0.001 between non-smokers and smokers. (**c**) Expression of *ACE2 (left)*, *TMPRSS2 (middle)*, and *CTSL (right)* in TCGA data for non-smokers and smokers with lung squamous cell carcinoma, lung adenocarcinoma, cervical cancer, bladder urothelial carcinoma or esophageal carcinoma split into age groups: below 40 years (n = 68), between 40 and 60 years (n = 328), above 60 years (n = 477). Wilcoxon signed rank tests were performed and significant differential expression between groups was labelled *p < 0.05; **p < 0.01 between non-smokers and smokers.

In the TCGA dataset, across 33 cancer types (n = 12,736), the sex annotations were also evaluated. There was consistent, significant upregulation of *ACE2, TMPRSS2*, and *CTSL* in males compared to females (Fig. 4a; *p* < 0.0001).

Notably, a number of the cancers included in this study have a strong association with smoking such as colon, lung, esophagus, bladder, and cervical cancers. Recall, all of these smoking-related cancers exhibited above average expression of *ACE2* and *TMPRSS2* amongst the 20 cancer types analyzed and the rank expression of *ACE2* was increased in these malignant tissues compared to their normal tissue counterparts (Fig. 2). Smoking status TCGA annotations were available for 4 out of 5 smoking-related cancer sites, including lung, esophagus, bladder, and cervix for 873 samples. Within these data, smokers demonstrated significantly upregulated expression of *CTSL* (Fig. 4b). On further categorization of smoking by age, it was identified that this smoking-related increase in *CTSL* was most notable amongst the older patients i.e. for those aged 40–60 (*p* < 0.01), and > 60 years (*p* < 0.05) (Fig. 4c).

### Radiotherapy transiently upregulates viral entry gene expression

**Figure 5:**
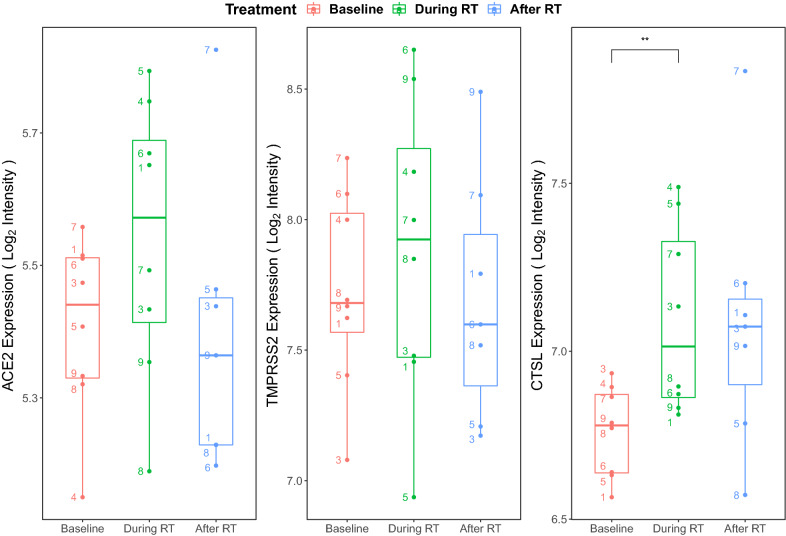
Effects of radiotherapy on ACE2, TMPRSS2, and CTSL expression. Expression of *ACE2 (left)*, *TMPRSS2 (middle)*, and *CTSL (right)* in buccal mucosa before, during, and after radiotherapy (RT) among 8 tonsillar squamous cell carcinoma patients from the GSE103412 data set. The probe intensity for each gene was log_2_ transformed and visualised for each treatment group to present normalized gene expression values. Paired t-tests were performed between adjacent time points and significant differential expression between groups was labelled. The numbers denote each of the 8 individual patient’s data points. Of note, patient #2′s data was not available for download from the Gene Expression Omnibus (GEO) database. **p < 0.01 between baseline and during radiotherapy.

The impact of radiotherapy on expression of SARS-CoV-2 entry factors is currently unclear. Marcussen et al.^33^ had previously evaluated the effects of radiotherapy on oral mucosal tissues. As the oral cavity is the entry passage for both the gastrointestinal and respiratory tracts, it provides a clinically relevant site to interrogate viral entry. In the Marcussen et al.^33^ study, 5-mm biopsies of buccal mucosa were collected before, during (after day 7 of radiotherapy), and after radiotherapy (20 days post-radiotherapy) in eight patients with tonsillar squamous cell carcinoma. Using this publicly available data set (GSE103412), we investigated how radiation could modulate the expression of *ACE2*, *TMPRSS2*, and *CTSL* in these samples. Figure 5 outlines the temporal expression patterns of these three genes. There were consistent trends towards elevation of gene expression during radiotherapy for all viral entry genes (*ACE2*: *p* = 0.17; *TMPRSS2*: *p* = 0.34; *CTSL*: *p* = 0.008). Post-radiotherapy, there was a decrease of *ACE2* and *TMPRSS2* below baseline levels, but a trend towards persistent elevation of *CTSL*.

**Figure 6:**
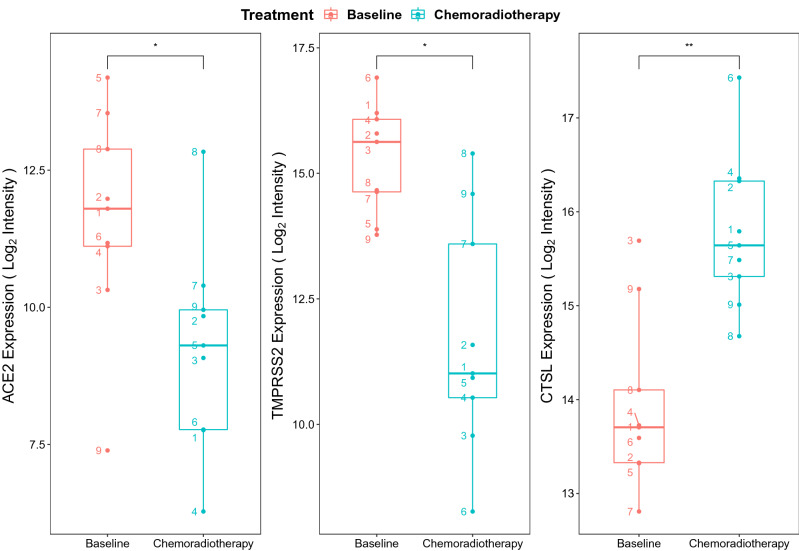
Effects of chemoradiotherapy on ACE2, TMPRSS2, and CTSL expression. Expression of *ACE2 (left)*, *TMPRSS2 (middle)*, and *CTSL (right)* in rectal tissue before and after chemoradiotherapy with 50 Gy in 25 fractions over 5 weeks plus capecitabine 825 mg/m^2^ twice daily during radiotherapy from the GSE15781 data set (n = 9 patients). The probe intensity for each gene was log_2_ transformed and visualised for each treatment group to present normalized gene expression values. Paired t-tests were performed between time points and significant differential expression between groups was labelled. The numbers denote each of the 9 individual patient’s data points. *p < 0.05. **p < 0.01 between baseline and chemotherapy groups.

With the addition of oral chemotherapy to radiotherapy, similar results were observed. Snipstad et al*.*^34^ (GSE15781) evaluated the RNA expression of nine patients’ rectal cancer tissue before and 4–6 weeks after pre-operative chemoradiotherapy of 50 Gy in 25 fractions over 5 weeks with capecitabine 825 mg/m^2^ administered twice daily during radiotherapy. We investigated the expression of these same viral entry genes, and observed that 4–6 weeks after chemoradiotherapy, there was a reduction of *ACE2* (*p* = 0.02) and *TMPRSS2* (*p* = 0.01), but continued increase in *CTSL* expression (*p* = 0.006) (Fig. 6).

### Radiotherapy transiently affects genes of host innate immunity

In addition to reducing viral entry, bolstering host viral defenses to limit viral replication and tolerating infection are key to mitigating impact of COVID-19; these defenses are mediated by host immunity. In the aforementioned buccal mucosa data set (GSE103412), the researchers simultaneously collected peripheral blood mononuclear cells (PBMCs). Gene set enrichment analysis of COVID-19-related genes^35^ expressed by PBMCs demonstrated that radiotherapy mediated significant downregulation across 153 GO and 3 KEGG pathways, including pathways for lymphocyte differentiation and signaling (Supplementary Fig. S1). Furthermore, eight of these pathways significantly overlapped with the 166 downregulated pathways during COVID-19 described by Xiong et al.^35^ (Supplementary Table S2). This highlights the potential dual targeting of radiation and COVID-19 infection on deregulating cell activation, recruitment of immune cells to sites of infection (via chemokine signaling), leading to weakened host defenses.

The impact of radiotherapy on mucosal immunity was further explored using the aforementioned GSE103412 dataset. During radiotherapy, type I IFN antiviral gene expression transiently declined, but promptly recovered after radiotherapy. This is shown with *SAMHD1* (*p* < 0.05), which is involved in suppression of viral replication (Supplementary Fig. S2). There was a similar pattern of response with *IFITM3;* however, changes in *IFITM3, MX1*, and *TRIM25* were not significant.

### Role of chemotherapy on viral defenses

**Figure 7:**
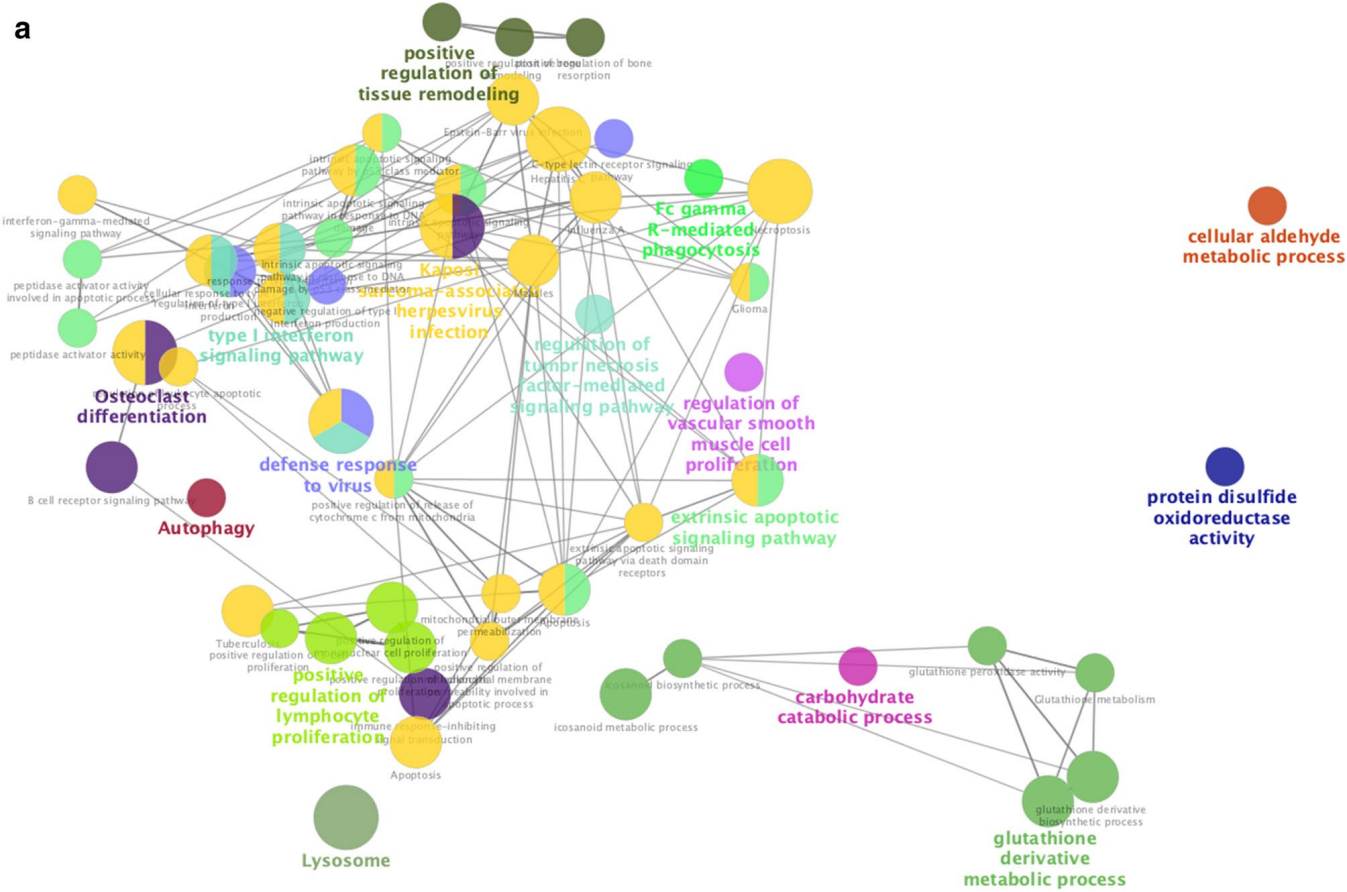

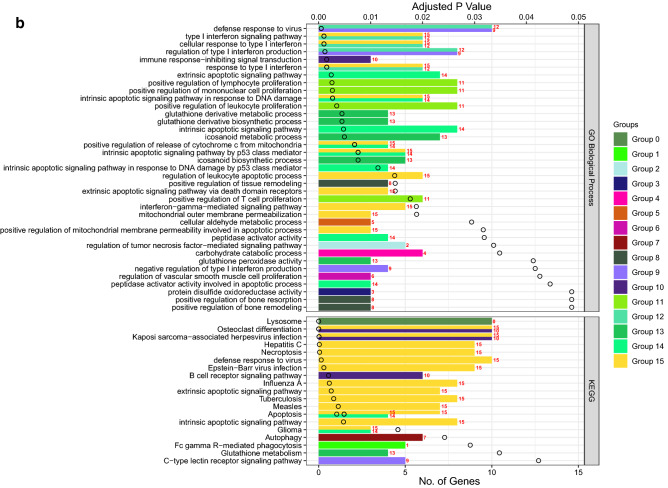
Functional analysis of upregulated genes during chemotherapy and COVID-19. Functional analysis using Cytoscape ClueGo App^57–59^ was performed on genes upregulated in peripheral mononuclear blood cells that are shared between chemotherapy treatment (GSE39324) and COVID-19^35^. The response to cyclophosphamide was analyzed in one patient with T-cell prolymphocytic leukemia, one patient with plasma cell leukemia, and eight patients with multiple myeloma. (**a**) Network of functional groups derived from ClueGO enrichment analysis showing 16 functional groups derived by Kappa statistics. The leading functional term for each group, defined by the term with the lowest adjusted p-value (Bonferonni step down method) within each cluster is coloured in bold. An overall q-value threshold of < 0.05 was used. Nodes are coloured by functional groups and the size of nodes are proportional to the q-value. (**b**) Bar chart of functional groups derived from ClueGO enrichment analysis. Terms are ordered by q-value, which is also presented on the upper y-axis; the q-value for each term is marked with a black circle. The lower x-axis defines the number of genes within each functional term. Terms are coloured by group, and the group is labelled to the right of each bar for clarity. The bar chart is also separated into 2 facets: GO Biological Process (top) and KEGG (bottom).

**Figure 8:**
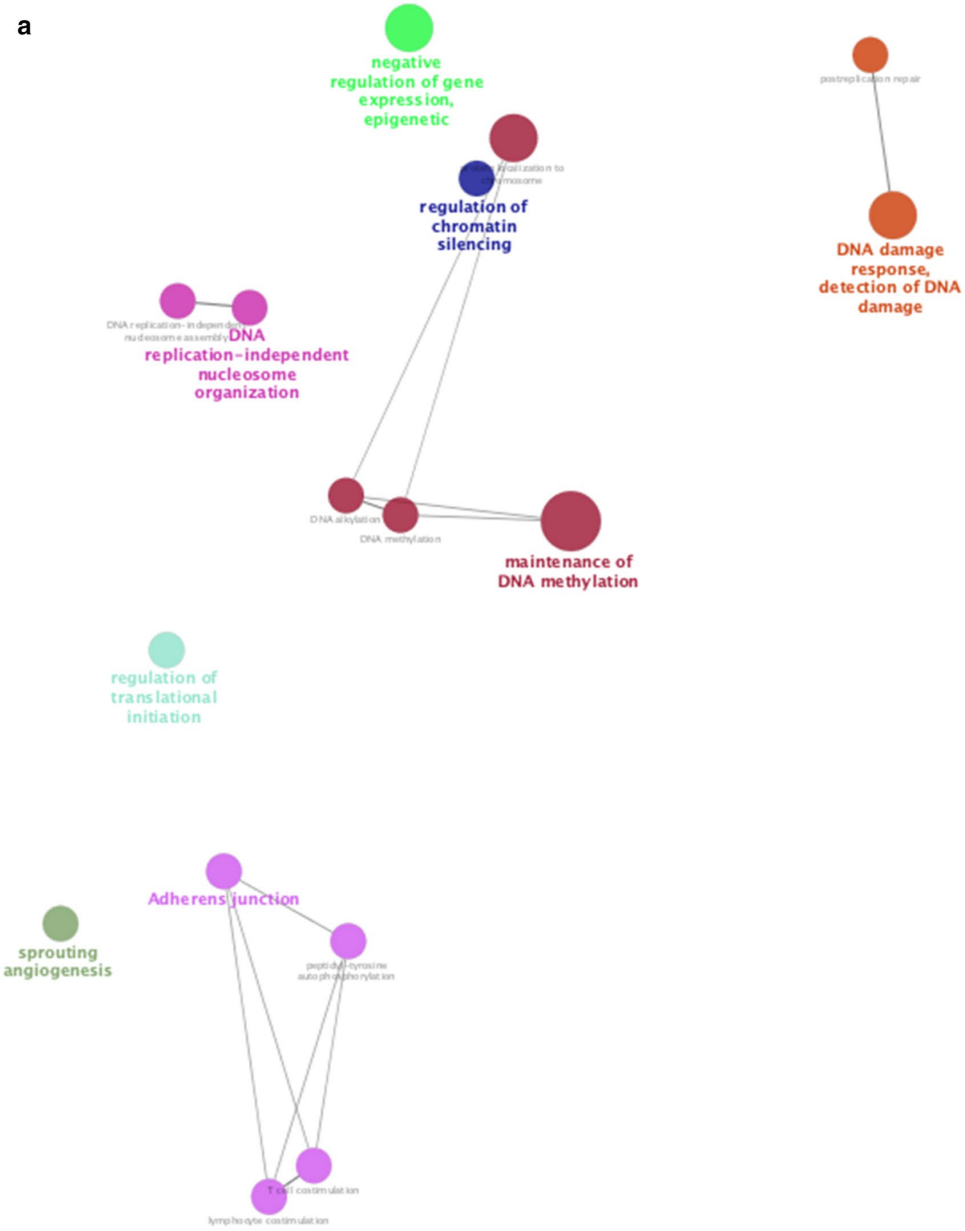

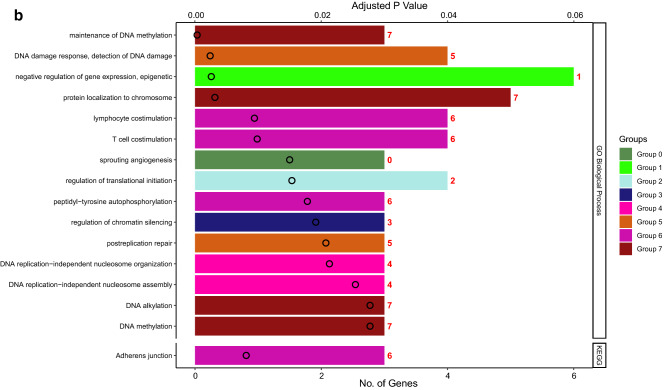
Functional analysis of downregulated genes during chemotherapy and COVID-19. Functional analysis using Cytoscape ClueGo App^57–59^ was performed on genes differentially downregulated by peripheral mononuclear blood cells that are shared between chemotherapy treatment (GSE39324) and COVID-19^35^. The response to cyclophosphamide was analyzed in one patient with T-cell prolymphocytic leukemia, one patient with plasma cell leukemia, and eight patients with multiple myeloma (as in Fig. 7). (**a**) Network of functional groups derived from ClueGO enrichment analysis. There were 16 functional groups derived by Kappa statistics. The leading functional term for each group, defined by the term with the lowest adjusted p-value (Bonferonni step down method) within each cluster is coloured in bold. An overall q-value threshold of < 0.05 was used. Nodes are coloured by functional group, and the size of nodes are proportional to q-value. (**b**) Bar chart of functional groups derived from ClueGO enrichment analysis. Terms are ordered by q-value, which is also presented on the upper y-axis. The q-value for each term is marked with a black circle. The lower x-axis defines the number of genes within each functional term. Terms are coloured by group and the group is labelled to the right of each bar for clarity. The bar chart is also separated into 2 facets: GO Biological Process (top) and KEGG (bottom).

We also assessed changes in RNA expression of PBMCs in response to cyclophosphamide chemotherapy, commonly used to treat both hematologic and solid malignancies. In GSE39324^36^, cyclophosphamide was administered to one patient with T-cell prolymphocytic leukemia, one patient with plasma cell leukemia, and eight patients with multiple myeloma. Gene set enrichment analysis identified upregulation of 14 GO pathways including neutrophil activation and degranulation, as well as downregulation of two GO pathways related to viral replication and cell death, which were shared pathways stimulated by both chemotherapy and COVID-19 infection (Supplementary Table S3, Figs. S3 and S4). Furthermore, functional analysis of all overlapping genes between chemotherapy treatment and COVID-19 further showed the shared upregulation of a number of pathways related to phagocytosis, innate viral defenses, type I IFN signaling, tumor-necrosis factor signaling, and innate cellular proliferation (Fig. 7). There was also a downregulation of regulatory pathways relating to DNA integrity, replication, and gene expression as well as regulation of lymphocyte stimulation (Fig. 8). Collectively, these data support the potential contributions of chemotherapy in targeting pathways deregulated during COVID-19 infection.

### Correlation of expression of viral response genes and COVID-19 severity

Using *CTSL* as a marker for viral entry gene expression, it was compared to the expression of type I IFN antiviral response genes (*IFITM3, MX1, SAMHD1*, and *TRIM25*) in GSE157103, which includes a large transcriptomic dataset from hospitalized COVID-19 patients requiring non-ICU care (50), ICU-level care (50), as well as 26 control patients without COVID-19. With non-severe (non-ICU) COVID-19, there was increased expression of the viral entry gene (*CTSL* (*p* < 0.05)) and increased antiviral response genes (*MX1* (*p* < 0.0001), *SAMHD1* (*p* < 0.0001), *TRIM25* (*p* < 0.01), *IFITM3* (ns)). During severe (ICU-level) COVID-19, three of the four antiviral response genes were no longer upregulated (Supplementary Fig. S5).

On further analysis, correlation between expression of viral entry genes and antiviral response genes was well-preserved during non-severe COVID-19, but was less prominent during severe COVID-19 (Supplementary Fig. S6). A multiple linear regression model was fitted to determine the statistical significance of the relationship between viral entry gene *CTSL* and viral defense genes. For patients with COVID-19 who were not admitted to ICU, there was a statistically significant relationship between viral defense genes and *CTSL* expression, the four predictors (*MX1, TRIM25 SAMHD1* and *IFITM3*) accounted for 65.56% of the variance (adjusted R^2^ = 0.656, p-value = 8.796^–11^). For patients with more severe symptoms (i.e. COVID-19 patients who were admitted to ICU), there was still a significant relationship between the four predictors and CTSL expression; however, these only accounted for 35.78% of the variance (adjusted R^2^ = 0.3578, p-value = 7.086^–05^). There was no significant association between viral response genes and viral entry genes in control patients who did not have COVID-19.

Finally, suppression of the viral response genes (*MX1* (*p* < 0.05), *SAMHD1* (*p* < 0.001), *IFITM3* (ns)) occurred more in severe ventilation-associated COVID-19 cases compared to non-ventilated cases (Supplementary Fig. S7). Consistent with prior analysis (Supplementary Fig. S5) however, *TRIM25* remains elevated in both severe ICU COVID-19 and ventilation-associated cases.

### Correlation of gene and protein expression

Next, we compared the similarity of gene and protein expressions of the viral entry factors. Unfortunately, expression of ACE2, TMPRSS2, and CTSL were not present in the reverse-phase protein arrays on The Cancer Genome Atlas (TCGA) database. However, immunohistochemistry-based malignant expression of ACE2, TMPRSS2, and CTSL was present in the Human Protein Atlas (https://www.proteinatlas.org/). Top expressing tissues were similar on gene and protein levels for ACE2 (colorectal, kidney, pancreas, and stomach), TMPRSS2 (prostate and pancreatic cancers), and CTSL (renal and skin cancers) (Fig. 2 and Supplemental Fig. S8). Cancers with lower gene expression levels were undetectable at the protein level.

## Discussion

As the etiologic agent of the ongoing COVID-19 pandemic, SARS-CoV-2 is a novel coronavirus that first emerged in Wuhan City, Hubei province, China, in December 2019. In the eight months since the outbreak first began in China, SARS-CoV-2 has rapidly spread, affecting 191 countries and territories yielding over 54.8 million confirmed cases and 1.3 million deaths as of November 16, 2020^37^.

ACE2^38^, TMPRSS2^39^, and cathepsins^11,40^ have been identified as key receptor and proteases interacting with the SARS-CoV-2 spike protein for viral entry, respectively. Thus far, expression of these genes has mostly been examined in the context of lung tissues^41^, identifying higher expression of ACE2 in lung alveolar type II cells^13^, and higher expression of TMPRSS2^41^ and cathepsin^42^ within a subset of ACE2-positive cells. This supports the consistent observation that the respiratory tract is a primary site of entry for the SARS-related coronaviruses including SARS-CoV-2. Less is known however; regarding their expression in other normal and malignant tissues that may also facilitate viral entry.

Recently, Zou et al*.*^13^ analyzed gene expression datasets to construct a risk map of tissues susceptible to SARS-CoV-2 infection based on *ACE2* expression. Our analysis further examined the gene expression of critical SARS-CoV-2 entry factors that have been confirmed thus far, including *ACE2, TMPRSS2*, and *CTSL*, across 20 normal tissues. Our data support the respiratory, gastrointestinal and genitourinary tracts as possible routes more susceptible to viral infection compared to other tissues (Fig. 2). Expression of these entry factors in gastrointestinal tissues, including the esophageal upper epithelial and gland cells as well as intestinal epithelial cells such as absorptive enterocytes of the ileum and colon, have similarly been corroborated by other groups^42,43^.

Compared to normal lung tissue, malignant lung tissue has been found to have elevated expression of *ACE*2^44^. In our analysis, elevation of *ACE2, TMPRSS2, and CTSL* in cancer *vs*. normal tissue was observed in many of the tissues examined, including many non-respiratory tract tissues (Fig. 2). Our analysis supports the observations that respiratory (lung) and gastrointestinal (esophageal) malignancies have been the most frequent cancer types described for patients infected with SARS-CoV-2, comprising approximately 25% and 14% of infected patients, respectively^45^. It is noted however, that the rank expression of these viral entry genes did not always increase from the normal to malignant states. This separate regulation of the viral entry genes has also been cited in other literature^44^. Whether an increase in all genes is necessary for increased susceptibility in cancer patients remains an area requiring more investigation.

Subsequently, an analysis of the effects of patient factors such as age, sex, and smoking were examined in this current study. Specifically, we noted decreased expression of viral entry genes among pediatric compared to adult samples (Fig. 3), supporting previous observations of decreased susceptibility of COVID-19 amongst children^46^. We additionally noted that males exhibited greater expression of entry factors compared to females (Fig. 4a), which is consistent with sex differences in mortality from COVID-19^47^. Prior evidence also showed that smoking has been observed to upregulate *ACE2* expression^48^, and has higher prevalence in cancer compared to non-cancer patients (22% *vs*. 7%)^2^. Our analysis additionally identified upregulation of CTSL expression with smoking, particularly prominent in those older than 40 years (Fig. 4b,c).

Additionally, our treatment analysis suggests transiently increased expression of viral entry genes during radiotherapy based on two datasets (GSE103412, GSE15781). In the radiotherapy alone dataset (GSE103412), samples were taken after day 7 of radiotherapy and 20 days post-radiotherapy. There was an early rise in gene expression (as early as day 7) and resolution of gene expression (as early as 20 days post-radiotherapy for *ACE2/TMPRSS2*) (Fig. 5). In the chemoradiotherapy dataset (GSE15781), samples were obtained prior to treatment and 4–6 weeks post-treatment. The non-elevated levels of the *ACE2/TMPRSS2* at 4–6 weeks post-treatment were consistent with the radiotherapy dataset as was the sustained elevation of *CTSL* (Fig. 6). Together, from these two datasets, it appears that *ACE2/TMPRSS2* have transient elevation with cancer treatments that resolve in less than 20 days post-treatment, while *CTSL* exhibits a prolonged elevation at for at least 4–6 weeks post-treatment.

Furthermore, both radiotherapy and chemotherapy targeted pathways of innate immunity that are also deregulated in COVID-19. Mechanisms of resistance against viral infection include early non-specific responses, and late cell-mediated and humoral immune responses during the stage two acute disease (pneumonia) phase to allow recovery in COVID-19 patients^49^. In patients with active co-morbidities that induce an immunodeficient state however, their impaired immune system does not effectively combat the acute viral pneumonia, thereby predisposing progression into the stage three severe or critical COVID-19^49^. In this study, radiotherapy appears to be suppressing expression of some type I IFN response genes (Supplementary Fig. 2); type I IFN responses are required initially to limit the viral burden of disease^50^. Furthermore, leukopenia and lymphopenia, which have been consistently noted in 32% and 82% of SARS-CoV-2-infected cancer patients, respectively^45^. Conversely, chemotherapy (cyclophosphamide) stimulates neutrophil activation and degranulation (Supplementary Fig. 3), which have been noted to occur in the hyperinflammatory response of severe COVID-19 cases^12,14,18^. These data are consistent with studies that have demonstrated that SARS-CoV-2-infected cancer patients have more severe events if they had a recent history of treatment with chemotherapy, targeted therapy, radiotherapy, or immunotherapy^45^. Understanding how to modify the timing of radiation and chemotherapy to avoid exacerbating the innate immunity dysregulation observed with COVID-19 may be important to guide clinical management of cancer patients requiring treatment during active infection.

Limitations of this analysis should be noted however, as the variables collected in the radiotherapy, chemotherapy, and COVID-19 datasets are confined to the original study design with missing data on the baseline immune characteristics of the cancer patients in the former studies, and lack of cancer characteristics in the COVID-19 dataset. Ideally, a single prospective dataset of cancer patients with and without COVID-19 with temporal transcriptomic profiles of the patients before, during, and after radiotherapy/chemotherapy treatments would be most useful in the future. Additionally, it is noted that gene-to-protein translation could not be validated for all tissue types, since these studies were limited by the number and types of antibodies used, as well as the number of available patient samples.

In our study, we explored the potential biological rationale behind increased COVID-19 among cancer patients using published literature and an analysis of large-scale genome-wide transcriptional data of normal and malignant tissues from human adults and children. Evidence supports a mechanistic relationship underlying the increased infectivity and severity of COVID-19 in oncology patients based on increased viral entry and reduced host resistance. To mitigate risks, shortening treatment schedules or modifying the sequencing or timing of cancer treatments may be helpful if oncologically safe to do so. Close monitoring of infectious symptoms during treatment may also be warranted. We do note some recent reassuring reports, including one from the UK, that have not observed any immediate deleterious effects of chemotherapy on COVID-19 patients^51^. As the global experience with COVID-19 continues to accumulate, the community will collectively achieve a greater understanding of the interactions between cancer therapy with this novel disease. Continued investigation of the biology of this disease as well as larger, comprehensive epidemiological studies will be important to further elucidate the complex relationship between COVID-19 and patients with cancer.

## Methods

### Data collection

The UCSC XenaTools platform^52^ was used to download gene expression data from TCGA, TARGET and GTEx. The UCSC XenaTools platform was also used to download clinical annotations for TCGA data. The GEOquery R package^53^ was used to download gene expression data along with associated clinical data from the GEO database for GSE103412 and GSE15781. Genes of interest, which were found to be previously differentially regulated in COVID-19, were obtained from the data sets along with differentially regulated GO and KEGG pathways. Further gene sets were obtained from previous chemotherapy-focused studies: a list of differentially expressed genes was obtained from (GSE39324^36^).

### Statistical analysis: TCGA, GTEx and TARGET

Data from GTEx and TCGA were log_2_ transformed. The cohorts from GTEx and TCGA were matched based on anatomical site, in total there were 20 sites. Mean log_2_ TPM expression of *ACE2*, *TMPRSS2* and *CTSL* was calculated for each tissue for both databases. The tissues were then ranked using the R function rank, 1 being the highest expression, 20 the lowest. The ranks were visualized using ggplot2. Wilcoxon signed rank tests were performed to compare *ACE2*, *TMPRSS2* and *CTSL* expression for tissues of interest, normal tissues (GTEx) compared to cancerous tissues (TCGA). Overall expression of *ACE, TMPRSS2*, and *CTSL* was visualised for all tissues from TCGA and TARGET. Tukeys test was performed in R to determine significance.

### Differential expression analysis

The limma^54^ package was used to perform differential expression analysis on GSE103412 and GSE15781. Oral buccal mucosa data from GSE103412 was obtained for the initial analysis. Gene expression was visualised using ggplot2, depicting overall log_2_ probe intensity for all patients. Subsequent paired t-tests were performed on gene expression values for patients to determine whether gene expression was significantly impacted by treatment. For both GSE103412 and GSE15781, a linear model was fit for the 3 genes of interest using the limma package^54^ to calculate log_2_ fold change, the patient ID was used as a blocking factor, which accounts for within patient bias. Expression was compared between different time points (before, during, and after treatments). For the oral mucosa dataset, GSE103412, gene expression of ISGs (*MX1, SAMHD1, IFITM3,* and *TRIM25*) were also visualized using ggplot2, depicting overall log_2_ probe intensity for all patients, subsequent paired t-test were performed for on expression values and compared between different time points.

For comparative purposes, gene expression values for a large cohort, consisting of COVID-19 and control patients (GSE157103) were visualised using ggplot2. Patients were stratified into non-COVID-19 (n = 26), non-ICU care COVID-19 patients (n = 50) and ICU-level care COVID-19 patients (n = 50). Subsequent Wilcoxon signed-rank tests were performed, and expression was compared between different groups. COVID-19 patients (n = 100) were also stratified into groups based on whether patients had been put on a mechanical ventilator (patients who had not been on mechanical ventilation (n = 58) and patients who had been on mechanical ventilation (n = 42)). Next, a differential expression analysis was performed on the peripheral mononuclear blood cell data from GSE103412. The data was a subset for specific genes of interest detailed in Xiong et al.^35^ where there were 13,745 out of 39,189 genes that overlapped in the gene expression matrix. The makeContrasts limma function was used to determine contrasts between treatment groups. A linear model was then fit to the data, and the contrasts.fit function was applied to identify genes with significant differential expression between samples prior to radiation treatment, *vs*. samples during radiation treatment. The empirical Bayes variance moderation method was applied via the eBayes function; the output was moderated t-statistics. The number of differentially expressed genes was extracted via the topTable function and an adjusted p value threshold < 0.05 was set, from which there were 243 significantly differentially expressed genes. Fold change cut-offs of 1.5 and -1.5 were implemented to determine up- and downregulated genes. In total, there were 65 significantly downregulated genes and 3 significantly upregulated genes that fit the criteria.

### Enrichment analysis

For GSE103412, enrichment analysis was performed on 65 significantly downregulated genes using clusterprofiler^55^. The enrichGO and enrichKEGG functions was used to infer supressed GO and KEGG pathways in patients during radiation treatment *vs*. prior treatment. The Benjamin & Hochberg^56^ adjusted p-value method was implemented, and a threshold of 0.05 was set, significantly supressed GO and KEGG pathways were thus obtained. These pathways were merged with known coronavirus pathways^35^ using R to obtain overlapping GO pathways.

Enrichment analysis was also performed on genes that were differentially expressed in 1–2 days of treatment with chemotherapy (GSE39324)^36^ *vs*. prior treatment using clusterprofiler^55^. The enrichGO and enrichKEGG function was applied with the Benjamin & Hochberg^56^ method enforced (threshold 0.05). The output was merged with known COVID-19 pathways using R to identify overlapping upregulated and downregulated GO and KEGG pathways.

Hypergeometric tests were performed using the inbuilt R function phyper, to determine whether the overlap between differentially regulated pathways in radiotherapy and chemotherapy versus differentially regulated COVID-19 pathways was deemed significant.

### Functional analysis

Functional Analysis using Cytoscape ClueGO App^57–59^ was performed on the overlapping differentially expressed genes by peripheral blood mononuclear cells from chemotherapy-treated (GSE39324)^36^ and COVID-19^35^ datasets. 135 upregulated and 112 genes downregulated were uploaded separately to ClueGO. The software uses Kappa statistics to link terms within a network, two-sided hypergeometric tests were used to define enrichment, the Bonferroni step down p value correction method was also implemented with a q-value threshold of ≤ 0.05.

### Sex analysis

All TCGA annotations which contained gender status (male/female) were analyzed for 33 cancer types (n = 12,736). Wilcoxon signed-rank tests were performed to determine the difference in expression for viral entry genes *ACE2, TMPRSS2*, and *CTSL*.

### Smoking analysis

TCGA data for five smoking related cancers (lung squamous cell carcinoma, lung adenocarcinoma, cervical cancer, bladder and esophageal) were extracted using the XenaTools platform, then filtered based on smoking status (n = 873). The remaining data consisted of two categories: ‘current smokers’ (smokers) or ‘lifelong non-smokers’ (non-smokers); values and definitions were obtained from the TCGA data dictionary (https://cdebrowser.nci.nih.gov/cdebrowserClient/cdeBrowser.html#/valueDomain). Analysis was further stratified by age. Wilcoxon signed-rank tests were performed to determine the difference in expression for viral entry genes *ACE2, TMPRSS2* and *CTSL*.

### Correlation analysis between viral response and viral entry gene CTSL

For a large cohort of COVID-19 (n = 100) and non-COVID-19 patients (n = 26), patients were stratified into groups based on severity: ‘COVID-19-ICU’ (n = 50), ‘COVID-19-Non-ICU’ (n = 50) and ‘Non-COVID-19′ (n = 26). Spearman’s rank correlations were performed to determine the strength of the relationships between viral entry gene *CTSL* and viral response genes *MX1*, *SAMHD1*, *IFITM3* and *TRIM25*. A multiple linear regression model was also fitted using the lm function in R to determine whether the relationship between viral defense genes and *CTSL* expression was statistically significant.

### Protein analysis of viral entry genes

Protein expression plots were exported from the human protein atlas (https://www.proteinatlas.org/)^60^ to determine whether there is evidence of protein expression of viral entry genes. ACE2 was probed by two antibodies (HPA000288 and CAB026174), TMPRSS2 was probed by one antibody (HPA035787), and CTSL was probed by one antibody (CAB000459).

## Supplementary Information

Supplementary Information

## Data Availability

All data is available on the University of California Santa Cruz (UCSC) Xena platform and the Gene Expression Omnibus (GEO) repository.
