# Supplementary material for: Elevation in viral entry genes and innate immunity compromise underlying increased infectivity and severity of COVID-19 in cancer patients
Source: Sci Rep. 2021 Feb 25;11:4533. doi: 10.1038/s41598-021-83366-y (PMC7907391; doi:10.1038/s41598-021-83366-y)
Supplement: Supplementary file 1 — Supplementary Information [file 41598_2021_83366_MOESM1_ESM.docx]

**Supplementary Information for:**

**Elevation in Viral Entry Genes and Innate Immunity Compromise Underlying Increased Infectivity and Severity of COVID-19 in Cancer Patients**

**Jennifer Yin Yee Kwan^1-2*^, Liang-Tzung Lin^3-4*^, Rachel Bell^5*^, Jeffrey P. Bruce^5^, Christopher Richardson^6-7^, Trevor J. Pugh^5,8^, Fei-Fei Liu^1-2,5,9-10^**

^1^Department of Radiation Oncology, University of Toronto, Toronto, Ontario, Canada

^2^Institute of Medical Science, University of Toronto, Toronto, Ontario, Canada

^3^Department of Microbiology & Immunology, Taipei Medical University, Taipei, Taiwan

^4^Graduate Institute of Medical Sciences, College of Medicine, Taipei Medical University, Taipei, Taiwan

^5^Princess Margaret Cancer Centre, Toronto, Ontario, Canada

^6^Department of Microbiology & Immunology, Faculty of Medicine, Dalhousie University, Halifax, Nova Scotia, Canada

^7^Department of Pediatrics, Faculty of Medicine, Dalhousie University, Halifax, Nova Scotia, Canada

^8^Ontario Institute for Cancer Research, Toronto, Ontario, Canada

^9^Department of Medical Biophysics, University of Toronto, Toronto, Ontario, Canada

^10^Radiation Medicine Program, Princess Margaret Cancer Centre, Toronto, Ontario, Canada

***** these three authors contributed equally

Corresponding Author

Dr. Fei-Fei Liu

Princess Margaret Cancer Centre

Radiation Medicine Program

700 University Avenue

Toronto, Ontario, Canada

M5G 2M9

Phone: (416)-946- 2123

Fax: (416)-946- 4586

Email: [Fei-Fei.Liu@rmp.uhn.on.ca](mailto:Fei-Fei.Liu@rmp.uhn.on.ca)

**Supplementary Table S1. Differences in expression of viral entry genes between normal and malignant tissues.**

Wilcoxon rank tests for RNA expression of *ACE2*, *TMPRSS2*, and *CTSL* between 20 matched normal and malignant human tissues from the Genotype-Tissue Expression Portal (GTEx, n= 4,744 Samples)[29] and The Cancer Genome Atlas (TCGA, n= 9,026 Samples)[30] respectively. P-values displayed in the Tables. *P<0.05. **P<0.01.

| **Malignant vs Normal Tissue** | **ACE2** | **TMPRSS2** | **CTSL** |
| --- | --- | --- | --- |
| Kidney Clear Cell Carcinoma  vs Kidney | 0.000002924 ** | 0.000000003539 ** | 0.000000000001852 ** |
| Bladder Urothelial Carcinoma  vs Bladder | 0.953 | 0.7857 | 0.009099 ** |
| Prostate Adenocarcinoma  vs Prostate | 0.000000000002537 ** | < 2.2e-16 ** | < 2.2e-16 ** |
| Colon Adenocarcinoma  vs Colon-Transverse | < 2.2e-16 ** | 0.8305 | 1.85E-11 |
| Colon Adenocarcinoma  vs Colon - Sigmoid | < 2.2e-16 ** | < 2.2e-16 ** | < 2.2e-16 ** |
| Stomach Adenocarcinoma  vs Stomach | < 2.2e-16 ** | 1.03E-07 | 1.16E-06 |
| Esophageal Carcinoma  vs Esophagus - Mucosa | 1.69E-01 | 0.8182 | 0.000000000000001614 ** |
| Esophageal Carcinoma  vs Esophagus - Muscularis | 0.0000000005865 ** | < 2.2e-16 ** | < 2.2e-16 ** |
| Esophageal Carcinoma  vs Esophagus - Gastroesophageal Junction | 0.00000001637 ** | < 2.2e-16 ** | < 2.2e-16 ** |
| Lung Adenocarcinoma  vs Lung | 0.0000000000004891 ** | 0.1026 | 2.34E-11 |
| Lung Squamous Cell Carcinoma  vs Lung | 1.80E-12 | < 2.2e-16 ** | < 2.2e-16 ** |
| Cervical & Endocervical Cancer  vs Cervix - Endocervix | 0.008961 ** | 0.2661 | 0.01096 * |
| Glioblastoma multiforme  vs Brain - Frontal Cortex (Ba9) | 0.0005897 ** | 0.0000000009381 ** | < 2.2e-16 ** |

**Supplementary Table S2. Pathways targeted by COVID-19 and radiotherapy treatment.**

Table of shared deregulated pathways in peripheral mononuclear blood cells by COVID-19 and radiotherapy. The Gene Ontology (GO) pathway, description, and significance of gene set enrichment are shown. Comparative gene set enrichment analysis with clusterProfiler[55] in R identified eight significantly downregulated pathways targeted by both COVID-19[35] and radiotherapy (GSE103412).

| **Pathway** | **Description** | **Regulatory Direction** | **q-value**  **(COVID-19)** | **q-value**  **(Radiotherapy)** |
| --- | --- | --- | --- | --- |
| GO:0001637 | G protein-coupled chemoattractant receptor activity | Down | 0.012 | 0.027 |
| GO:0004950 | Chemokine receptor activity | Down | 0.012 | 0.027 |
| GO:0009897 | External side of plasma membrane | Down | 0.033 | <0.00001 |
| GO:0009897 | External side of plasma membrane | Down | 0.016 | <0.00001 |
| GO:0016493 | C-C chemokine receptor activity | Down | 0.01 | 0.027 |
| GO:0019957 | C-C chemokine binding | Down | <0.001 | 0.027 |
| GO:0030155 | Regulation of cell adhesion | Down | 0.041 | <0.001 |
| GO:0035710 | CD4-positive, alpha-beta T cell activation | Down | 0.04 | 0.005 |

**Supplementary Table S3. Pathways targeted by COVID-19 and chemotherapy treatment.**

Table of shared deregulated pathways in peripheral mononuclear blood cells by COVID-19 and chemotherapy. The Gene Ontology (GO) pathway, description, and significance of gene set enrichment are shown. Gene set enrichment analysis was performed using the Benjamin and Hochberg[56] method (q<0.05). Fourteen significantly upregulated and two significantly downregulated pathways were targeted by both COVID-19[35] and chemotherapy (GSE39324).

| **Pathways** | **Description** | **Regulatory Direction** | **q-value**  **(COVID-19)** | **q-value**  **(Chemotherapy)** |
| --- | --- | --- | --- | --- |
| GO:0002283 | Neutrophil activation involved in immune response | Up | 5.17E-05 | 3.47E-07 |
| GO:0002446 | Neutrophil mediated immunity | Up | 7.91E-05 | 3.47E-07 |
| GO:0005766 | Primary lysosome | Up | 0.00999 | 4.27E-06 |
| GO:0005775 | Vacuolar lumen | Up | 1.29E-05 | 7.18E-08 |
| GO:0031983 | Vesicle lumen | Up | 0.000409 | 0.000118 |
| GO:0034774 | Secretory granule lumen | Up | 0.000206 | 7.74E-05 |
| GO:0035578 | Azurophil granule lumen | Up | 0.00555 | 7.74E-05 |
| GO:0042119 | Neutrophil activation | Up | 3.50E-05 | 3.47E-07 |
| GO:0042582 | Azurophil granule | Up | 0.00999 | 4.27E-06 |
| GO:0043202 | Lysosomal lumen | Up | 0.00231 | 1.46E-05 |
| GO:0043312 | Neutrophil degranulation | Up | 4.52E-05 | 3.47E-07 |
| GO:0060205 | Cytoplasmic vesicle lumen | Up | 0.000408 | 0.000118 |
| GO:0070820 | Tertiary granule | Up | 0.00231 | 0.020775 |
| GO:0101002 | Ficolin-1-rich granule | Up | 0.000384 | 0.002593 |
| GO:0061136 | Regulation of proteasomal protein catabolic process | Down | 0.035 | 0.03189 |
| GO:1901216 | Positive regulation of neuron death | Down | 0.0348 | 0.0244 |

**a**

**b**

**Supplementary Fig. S1. Immune pathways significantly downregulated by radiotherapy.**

**a.** Gene Ontology (GO) Gene Set Enrichment Analysis of differentially expressed genes in peripheral mononuclear blood cells of 8 tonsillar squamous cell carcinoma patients comparing before and during radiotherapy time-points (GSE103412). 153 GO Pathways were significantly downregulated by radiotherapy. The top downregulated pathways are plotted by their adjusted p-values. BP: Biological Process. CC: Cellular Component. MF: Molecular Function.

**b.** Kyoto Encyclopedia of Genes and Genomes (KEGG) Gene Set Enrichment Analysis of differentially expressed genes in peripheral mononuclear blood cells of eight tonsillar squamous cell carcinoma patients comparing before and during radiotherapy time-points (GSE103412). Three KEGG Pathways were significantly downregulated by radiotherapy. These downregulated pathways are plotted by their adjusted p-values.

**Supplementary Fig. S2. Impact of radiotherapy on mucosal immunity.**

Expression of interferon-stimulated genes (*MX1*, *IFITM3*, *SAMHD1* and *TRIM25)* in buccal mucosa, before, during, and after radiotherapy (RT) among eight tonsillar squamous cell carcinoma patients from the GSE103412 data set. The probe intensity for each gene was log_2_ transformed and visualised for each treatment group to present normalized gene expression values. Paired t-tests were performed between adjacent time points, and significant differential expression between groups (p<0.01) was labelled. The numbers denote each of the eight individual patient’s data points. Of note, patient #2’s data was not available for download from the Gene Expression Omnibus (GEO) database; statistical significance is labelled as *P<0.05 between treatment groups.

**a**

**b**

**Supplementary Fig. S3. Immune pathways significantly upregulated by chemotherapy.**

**a.** Gene Ontology (GO) and **b.** Kyoto Encyclopedia of Genes and Genomes (KEGG) Gene Set Enrichment Analyses of differentially expressed genes in peripheral mononuclear blood cells of ten patients with hematologic malignancies (one patient with T-PLL, one patient with PCL, and eight patients with multiple myeloma) comparing before and 1-2 days after chemotherapy (GSE39324) using the Benjamin & Hochberg[56] method (q<0.05). 73 GO Pathways and 17 KEGG Pathways were significantly upregulated by chemotherapy. The top upregulated pathways are plotted by their adjusted p-values. BP: Biological Process. CC: Cellular Component. MF: Molecular Function.

**a**

**b**

**Supplementary Fig. S4. Immune pathways significantly downregulated by chemotherapy.**

**a.** Gene Ontology (GO) and **b.** Kyoto Encyclopedia of Genes and Genomes (KEGG) Gene Set Enrichment Analyses of differentially expressed genes in peripheral mononuclear blood cells of ten patients with hematologic malagnancies (one patient with T-PLL, one patient with PCL, and eight patients with multiple myeloma) comparing before and 1-2 days after chemotherapy (GSE39324) using the Benjamin & Hochberg[56] method (q<0.05). 114 GO Pathways and 2 KEGG Pathways were significantly downregulated by chemotherapy. The top downregulated pathways are plotted by their adjusted p-values. BP: Biological Process. CC: Cellular Component. MF: Molecular Function.

**Supplementary Fig. S5. Relationship between viral response genes and COVID-19 severity.** The GSE157103 dataset included 100 COVID-19 patients (50 ICU, 50 non-ICU) and 26 non-COVID-19 patients. Expression of *CTSL*, *MX1*, *TRIM25*, *SAMHD1* and *IFITM3* for non-COVID-19 controls, COVID-19 patients who were not admitted to ICU, and COVID-19 patients admitted to ICU is shown. Wilcoxon signed rank tests were performed, and statistical significance between groups was labelled. *P<0.05; **P<0.01;***P<0.001;****P<0.0001.

**Supplementary Fig. S6. Spearman rank correlations between viral entry and defense genes.** The GSE157103 dataset of 100 COVID-19 patients (50 ICU, 50 non-ICU) and 26 non-COVID-19 patients was used for this analysis. Spearmans rank correlations between viral defense genes expression (log_2_ TPM), and expression of viral entry gene *CTSL* (log_2_ TPM) in COVID-19 patients are shown. Plots are annotated with correlation coefficients (R) and p-values.

**Supplementary Fig. S7. Relationship between viral response genes and ventilator use with COVID-19.** Within the GSE157103 dataset, there were 58 COVID patients who had not been on mechanical ventilation *vs*. 42 who had been on mechanical ventilation. Expression of *CTSL*, *MX1*, *TRIM25*, *SAMHD1*, and *IFITM3* were stratified into the two groups are shown. Wilcoxon signed rank tests were performed, and statistical significance between groups was labelled. *P<0.05; **P<0.01;***P<0.001.

**a**

**
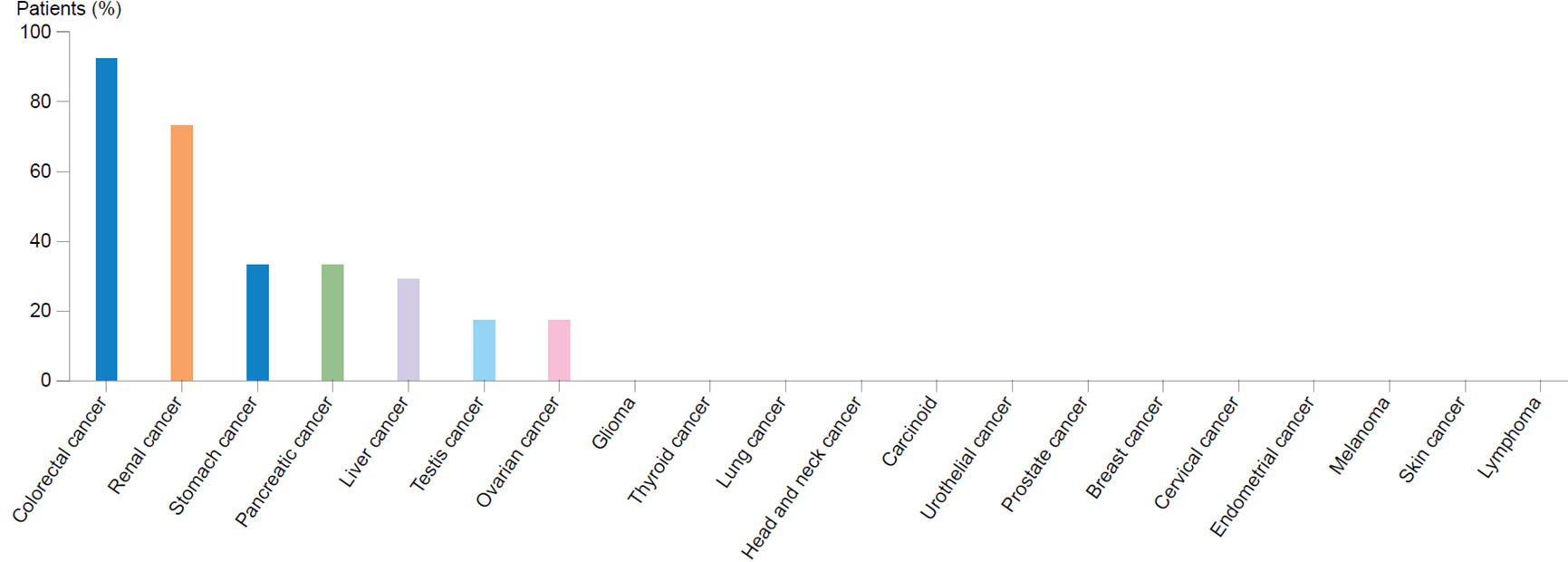
**

**b**

**
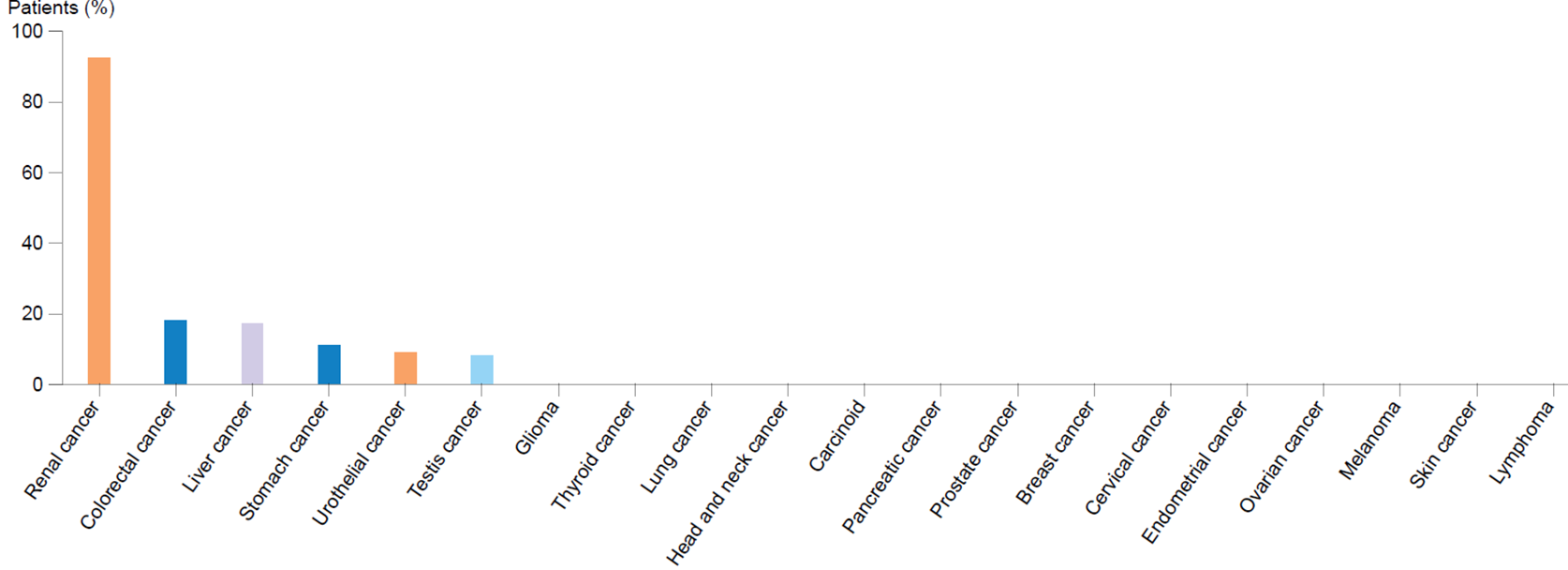
**

**c**

**
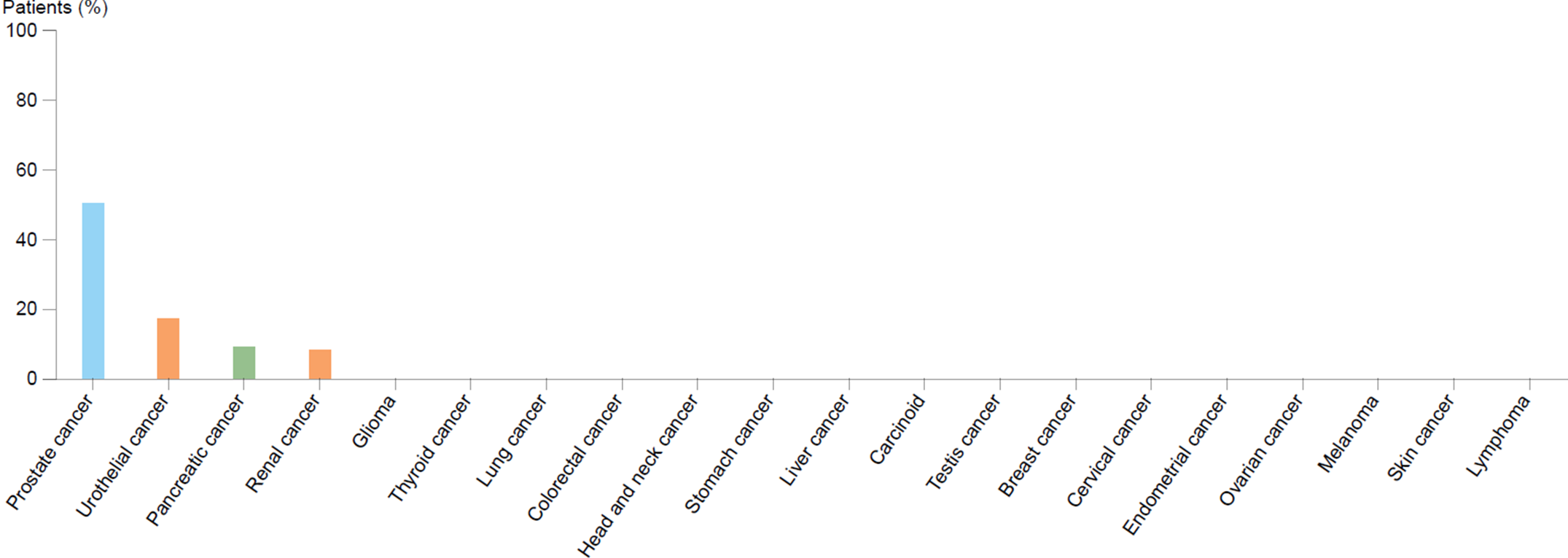
**

**d**

**
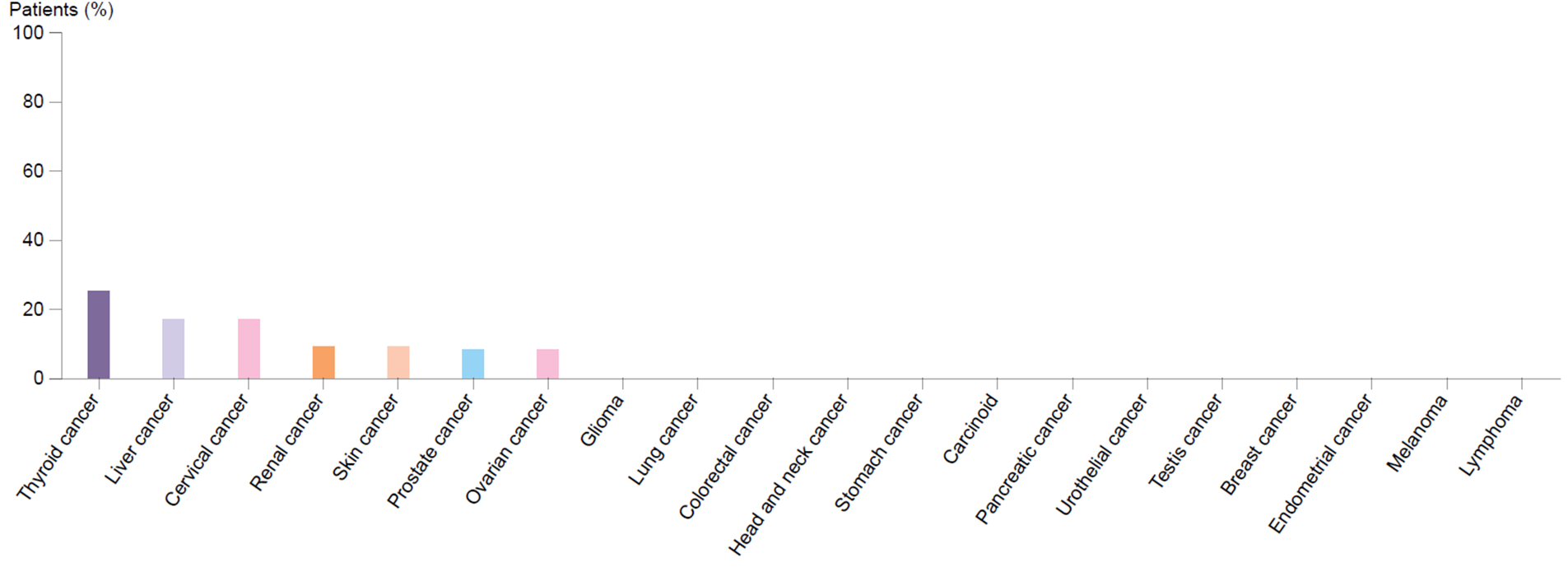
**

**Supplementary Fig. S8. Correlation of gene and protein expression in cancers.** Human Protein Atlas (<https://www.proteinatlas.org/>) immunohistochemistry (IHC) data show percentage of patients with various cancers who express ACE2, TMPRSS2 and CTSL in their tumour tissues. Image credit: Human Protein Atlas.

**a-b.** Cancers that showed medium to high expression of ACE2. **a.** HPA000288 antibody detected expression in colorectal (11 of 12 patients), renal (8 of 11 patients), stomach (4 of 12 patients), pancreatic (4 of 12 patients), liver (2 of 7 patients), testis (2 of 12 patients), and ovarian cancer (2 of 12 patients). **b.** CAB026174 antibody detected expression in: renal (11 of 12 patients), colorectal (2 of 11 patients), liver (2 of 12 patients), stomach (1 of 9 patients), urothelial (1 of 11), and testis cancer (1 of 12 patients). Other cancers exhibited low or no detection by the antibodies (4 to 12 patient samples were tested for those cancers). For details: <https://www.proteinatlas.org/ENSG00000130234-ACE2/pathology> (Protein Atlas version 19.3).

**c.** Cancers that showed medium to high expression of TMPRSS2 (antibody: HPA035787). Prostate (6 of 12 patients), urothelial (2 of 12 patients), pancreatic (1 of 11 patients), and renal cancer (1 of 12 patients). Other cancers exhibited low or no detection by the antibodies (3 to 12 patient samples were tested for those cancers). For details: <https://www.proteinatlas.org/ENSG00000184012-TMPRSS2/pathology> (Protein Atlas version 19.3).

**d.** Cancers that showed medium to high expression of CTSL (antibody: CAB000459). Thyroid (1 out 4 patients), liver (2 of 12 patients), cervical (2 of 12 patients), renal (1 of 11 patients), skin (1 of 11 patients), prostate (1 of 12 patients), and ovarian cancer (1 of 12 patients). Other cancers exhibited low or no detection by the antibodies (3 to 12 patient samples were tested for those cancers). For details: <https://www.proteinatlas.org/ENSG00000135047-CTSL/pathology> (Protein Atlas version 19.3).
